# Interruption of Lymph Flow Worsens the Skin Inflammation Caused by Saprophytic *Staphylococcus epidermidis*

**DOI:** 10.3390/biomedicines11123234

**Published:** 2023-12-06

**Authors:** Marta Cąkała-Jakimowicz, Anna Domaszewska-Szostek, Monika Puzianowska-Kuznicka

**Affiliations:** 1 Department of Human Epigenetics, Mossakowski Medical Research Institute, Polish Academy of Sciences, 02-106 Warsaw, Poland; adomaszewska@imdik.pan.pl; 2 Department of Geriatrics and Gerontology, Medical Centre of Postgraduate Education, 01-813 Warsaw, Poland

**Keywords:** lymphedema, lymph node, inflammation, immune cells, saprophytic flora, *Staphylococcus epidermidis*

## Abstract

Lymphedema is often complicated by chronic inflammation, leading to fibrosis, fat deposition, and inhibition of lymphangiogenesis. This study aimed to verify whether lymphedema itself or together with commensal bacterial flora infection contributes to the severity of local inflammation. Edema was induced by interruption of the lymph flow in the rat’s hind limb. Immune cell infiltrates were examined by flow cytometry and immunohistochemistry. Nine-day edema alone did not affect immune cell content in the skin but resulted in a decrease in CD4^+^ T helper lymphocytes and monocytes in the draining popliteal lymph nodes. In turn, local saprophytic *Staphylococcus epidermidis* infection of the edematous limb resulted in dense infiltrates of CD68^+^ macrophages and monocytes, MHC class II antigen-presenting cells, CD90^+^ stem cells, thymocytes, and immature B cells in the skin, accompanied by a simultaneous reduction in density of CD4^+^ T helper lymphocytes and monocytes, OX62^+^ dendritic cells, CD68^+^ macrophages and monocytes, HiS48^+^ granulocytes, CD90^+^ stem cells, thymocytes, and immature B cells in the draining popliteal lymph nodes. These results indicate that the combination of edema and saprophytic bacteria infection induces severe inflammation in the peripheral tissues and results in a delay of antibacterial protection processes in neighboring lymphatic organs.

## 1. Introduction

Lymphatics create networks of channels in peripheral tissues through which macrophages, lymphocytes, neutrophils, free antigens, solid particles, exosomes, and bacteria move together with afferent lymph to the draining lymph nodes [1,2,3]. Such migration is necessary to alleviate and resolve inflammation and for the development of peripheral tolerance [4]. Lymphatics eliminate macrophages involved in the regulation of inflammation and tissue infection but are also exploited by pathogenic microorganisms that colonize the host and spread systemically [5,6,7].

The skin is inhabited by saprophytic bacteria, the most common of which are cocci [8]. Commensals are not pathogenic as long as they inhabit their physiological niche, but injuries and abrasions create sites that can allow microorganisms to penetrate into deep tissues and commensals to become pathogenic. After penetrating the epidermis and entering deeper dermis layers, bacteria stimulate a local defense reaction [9,10]. Venous insufficiency, chronic inflammation, and damage to lymphatic vessels contribute to the development of chronic tissue swelling [11,12,13]. Notably, impaired patency of the lymphatic channels results in the local retention of microorganisms, which can continuously penetrate the skin, especially if it is damaged [13,14,15,16]. Microorganisms multiply in swollen tissues and trigger the host immune response [12,17,18,19]. Histological images of skin affected by lymphedema (a condition in which the drainage of lymph from the tissues is impaired and consequently stagnates) reveal infiltration of inflammatory cells, which play a key role in the pathology of the disease [20]. Patients with lymphedema exhibit a high concentration of lymphocytes in the peripheral lymph and an increased density of MHC class II presenting cells, Langerhans cells, macrophages, and granulocytes in the skin [15,21], as well as the presence of bacteria in deep tissues, tissue fluid, and lymph, mainly saprophytic staphylococci [16,19,22,23]. Chronic inflammatory changes lead to skin fibrosis and contribute to recurrent infections and even sepsis [9,12,17,22,24,25]. Fortunately, long-term administration of low doses of benzathine penicillin inhibits the growth of bacteria and prevents the recurrence of dermato-lymphangio-adenitis (DLA, episodic, recurrent inflammation of the skin, vessels, and lymph nodes in patients with lymphedema) [22,26,27,28,29].

The aims of the present study were to establish whether altered lymphatic drainage intensifies the immune reaction in the skin and popliteal lymph nodes upon local infection with saprophytic *Staphylococcus epidermidis* and to determine the immune cells participating in the response against this bacterium. We believe that our results will allow us to determine the common contribution of lymphedema and infestation with cutaneous commensals to chronic inflammation and its consequences.

## 2. Materials and Methods

### 2.1. Interruption of Afferent Lymphatics

*S. epidermidis* inoculation, popliteal lymph node isolation, and skin biopsies were performed as previously described [10]. Following the three Rs (replacement, reduction, and refinement) principle, to limit the number of animals, we obtained consent from the editor of our previous work (Cąkała-Jakimowicz et al., 2022 [10]) to use data regarding animals without infection and with intact lymphatics and animals infected with *S. epidermidis* and with intact lymphatics as the controls in the current study. Afferent lymphatics were visualized using 0.1 mL Patent Blue V (Merck KGaA, Darmstadt, Germany). Vessels visualized under the skin of the outer part of the lower leg were tied with sterile surgical threads near the tibia (day 1). On the next day (day 2), the appearance of lymphatic edema was observed on the dorsum of the foot; then, the animals were randomized into study groups. In the appropriate groups, injections of 0.9% NaCl or bacterial suspension were then started (Table 1, days 2–8). In all experimental groups, popliteal lymph nodes and paw skin were isolated on day 9 of the experiment (9 days after the ligation of the afferent lymphatics and 1 day after completion of a series of seven bacteria or saline injections).

### 2.2. Cell Isolation and Flow Cytometry

Cell isolation and flow cytometry are described in detail in Cąkała-Jakimowicz et al. [10]. Briefly, popliteal lymph nodes were weighed, the isolated cells were counted, their viability was assessed in a Bürker chamber, and the cells were then subjected to a standard antibody staining procedure. Popliteal lymph node cell phenotypes were assessed based on their positive staining for a given antigen using an LSR flow cytometer (Becton Dickinson, San Jose, CA, USA) and CellQuest Pro software v. 9.2 (Table 2, Supplementary Table S1).

### 2.3. Immunohistochemistry

This procedure was detailed previously [10]. Briefly, popliteal lymph nodes and skin fragments were frozen, cut into 5 µm sections, and mounted on poly-α-lysine-coated slides. After fixation, they were stained with antibodies (Table 2, Supplementary Table S2) and visualized using the LSAB 2 AP complex (Dako, Glostrup, Denmark) and a substrate for alkaline phosphatase or horseradish peroxidase. The slides were analyzed using a BX40 light microscope (Olympus, Hamburg, Germany). Positive (+) cells from four random fields of view (400×) were counted using ImageJ software v. IJ 1.46r.

### 2.4. Statistical Analysis

Statistical analysis was performed for four animal groups (two groups without afferent lymphatics ligation as described in Cakała-Jakimowicz et al., 2022 [10], and two groups with ligated afferent lymphatics) using one-way analysis of variance (ANOVA)/Kruskal–Wallis test followed by Tukey’s multiple comparison test and Dunn’s multiple benchmark test. Calculations were performed in Prism v. 7.04 (Graph Pad Software, Inc., San Diego, CA, USA). All data are reported as the mean of three independent experiments ± standard deviation. Statistical significance was set as *p* < 0.05.

## 3. Results

Because studies of patients with lymph stasis showed higher numbers of saprophytic bacteria in the skin and immunohistochemical studies revealed dense infiltrates of immune cells in the epidermis and dermis, suggesting an intensive antibacterial response [15], we decided to determine in a rat model how the disturbance of lymph flow affects the response of the immune system to massive infection with the most common cutaneous commensal, *S. epidermidis*. We compared animals massively infected with *S. epidermidis* and with lymph stasis with uninfected animals with lymph stasis. To obtain more complete data, we also compared lymph stasis groups to previously described groups with proper lymph flow [10].

### 3.1. Lymph Node Mass and Cell Number

**Figure 1:**
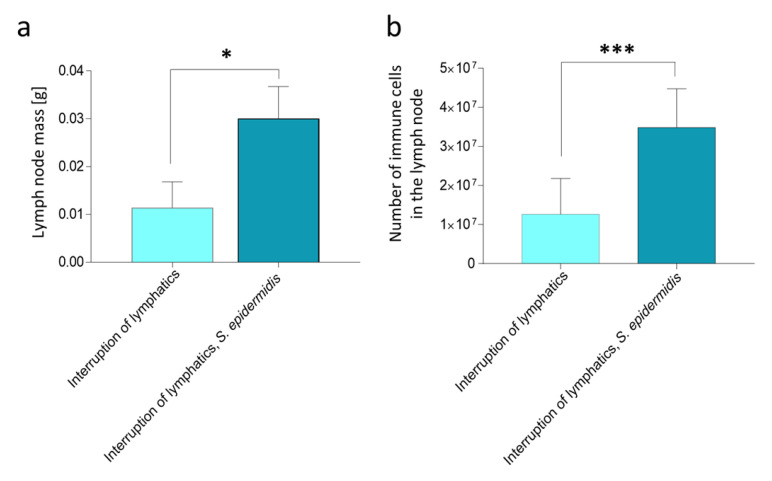
Rat popliteal lymph node mass and cell number on day 9 of the experiment, after 7 days of daily *S. epidermidis* or 0.9% NaCl administration. (**a**) Changes in lymph node mass (g). (**b**) Changes in the number of immune cells in the lymph node. Interruption of lymphatics: lymph stasis and 7 × 0.9% NaCl injection. Interruption of lymphatics, *S. epidermidis:* lymph stasis and 7× infection with *S. epidermidis.* Results are presented as mean ± standard deviation. * *p* < 0.05, *** *p* < 0.001.

On day 9 of the experiment, in the popliteal lymph nodes of animals with limb edema and massively infected with *S. epidermidis*, we observed a significant increase in the node mass and number of cells compared to uninfected animals with edema (*p* < 0.05 and *p* < 0.001, respectively) (Figure 1). Further comparison with the previously described animals with normal lymph flow [10] showed that 9-day lymph stasis itself did not affect the mass or number of cells in the popliteal lymph nodes. Moreover, the weight of nodes and the number of cells increased to the same extent upon *S. epidermidis* infection regardless of the lymph flow status.

### 3.2. Quantitative Changes in Cell Subpopulations in the Lymph Node

**Figure 2:**
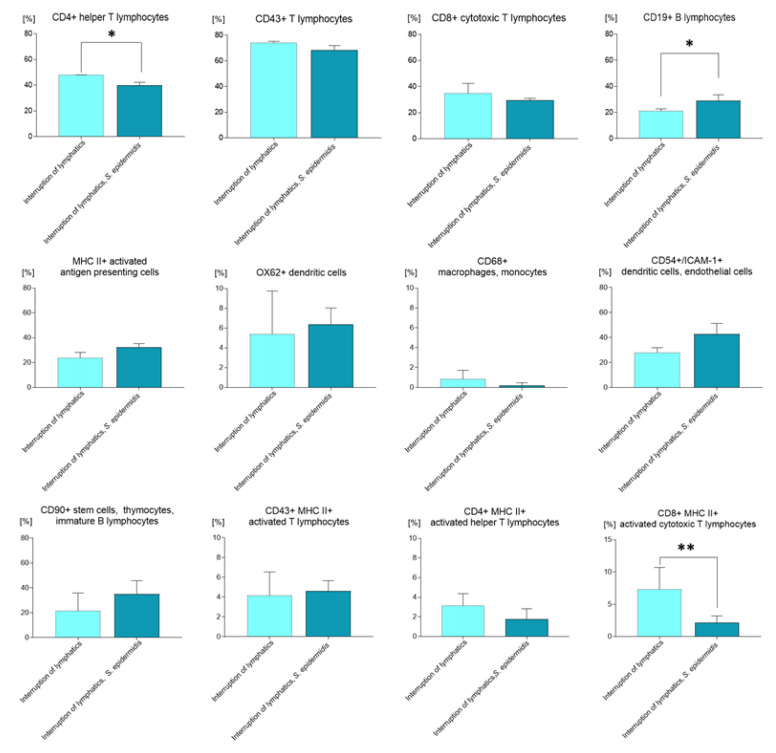
Changes in cell subpopulations of the popliteal lymph nodes assessed with flow cytometry on day 9 of the experiment, after 7 days of daily *S. epidermidis* or 0.9% NaCl administration. Interruption of lymphatics: lymph stasis and 7 × 0.9% NaCl injection. Interruption of lymphatics, *S. epidermidis:* lymph stasis and 7 × infection with *S. epidermidis.* Results are presented as mean ± standard deviation. * *p* < 0.05, ** *p* < 0.01.

Cytometric analysis revealed that the mere ligation of afferent lymphatics in animals injected with 0.9% NaCl resulted in a significantly lower percentage of CD4^+^ T helper lymphocytes and monocytes in the draining lymph nodes compared to the control group with normal lymph flow also injected with saline (*p* < 0.05). In contrast, we observed a significantly higher amount of CD4^+^ cells in the nodes of animals with ligated lymphatics and administered saline compared to the group with ligated lymphatics and massively infected with *S. epidermidis* (*p* < 0.05) (Figure 2). Moreover, in the group with affected lymph flow, we observed a significantly lower percentage of CD19^+^ B lymphocytes compared to the groups infected with *S. epidermidis*, both those with limb edema and those with normal lymph flow (both *p* < 0.05); a lower percentage of dendritic, endothelial, and ICAM-1^+^ cells (CD54^+^) compared to the group infected with *S. epidermidis* and with normal lymph flow (*p* < 0.05); and a higher percentage of CD8^+^ MHC II^+^ activated cytotoxic lymphocytes compared to animals with normal lymph flow administered saline or to animals with impaired lymph flow and infected with *S. epidermidis* (*p* < 0.05 and *p* < 0.01, respectively).

In animals with limb edema and infected with *S. epidermidis*, we observed a significantly lower percentage of CD4^+^ T helper lymphocytes and monocytes compared to the two groups administered saline, those with normal lymph flow, and those with afferent lymphatic ligation (*p* < 0.001 and *p* < 0.05, respectively). In these animals, we also detected a lower percentage of CD4^+^ MHC II^+^ activated T helper lymphocytes compared to the group with normal lymph flow without infection (*p* < 0.05), a lower percentage of CD8^+^ MHC II^+^ activated T cytotoxic lymphocytes compared to animals with limb edema and injected with saline (*p* < 0.01), and a significantly higher percentage of CD19^+^ B lymphocytes compared with the group with limb edema administered saline (Figure 2) (*p* < 0.05).

**Figure 3:**
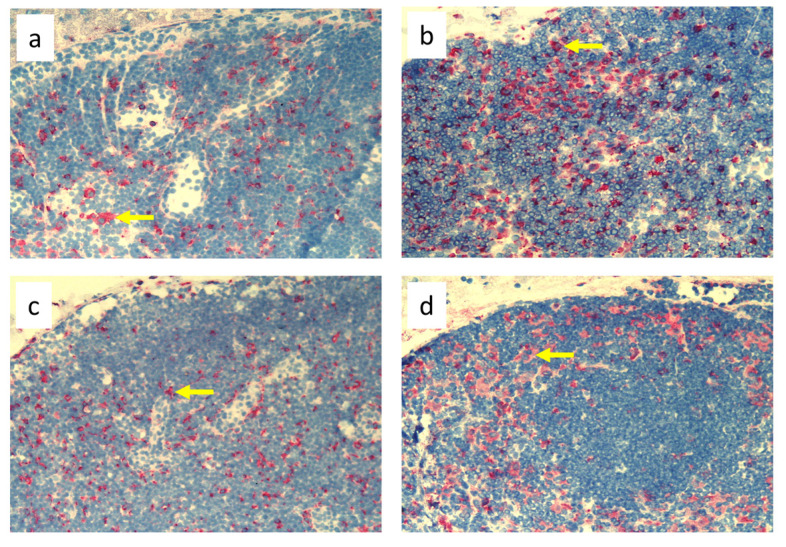
Popliteal lymph nodes stained with antibody against macrophages and monocytes (CD68^+^) on day 9 of the experiment. (**a**). Normal node, (7 × 0.9% NaCl). (**b**). Seven-times infection with *S. epidermidis*. (**c**). Ligation of afferent lymphatics and 7 × 0.9% NaCl. (**d**). Ligation of afferent lymphatics and 7× infection with *S. epidermidis*. Arrows indicate positively stained cells. Magnification, 200×. (**a**,**b**). as in Cąkała-Jakimowicz et al., 2022 [10], with permission.

**Figure 4:**
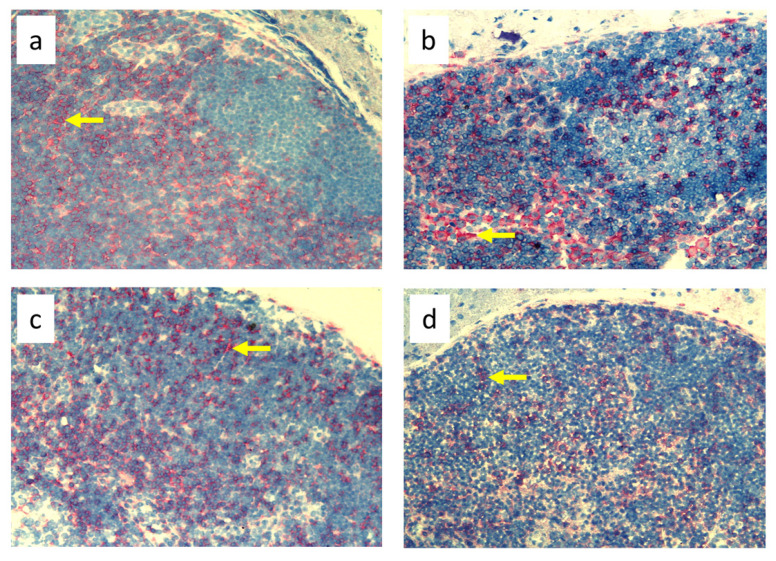
Popliteal lymph nodes stained with antibody against T helper lymphocytes and monocytes (CD4^+^) on day 9 of the experiment. (**a**). Normal node (7 × 0.9% NaCl). (**b**). Seven-times infection with *S. epidermidis*. (**c**). Ligation of afferent lymphatics and 7 × 0.9% NaCl. (**d**). Ligation of afferent lymphatics and 7× infection with *S. epidermidis*. Arrows indicate positively stained cells. Magnification, 200×. (**a**,**b**) as in Cąkała-Jakimowicz et al., 2022 [10], with permission.

**Figure 5:**
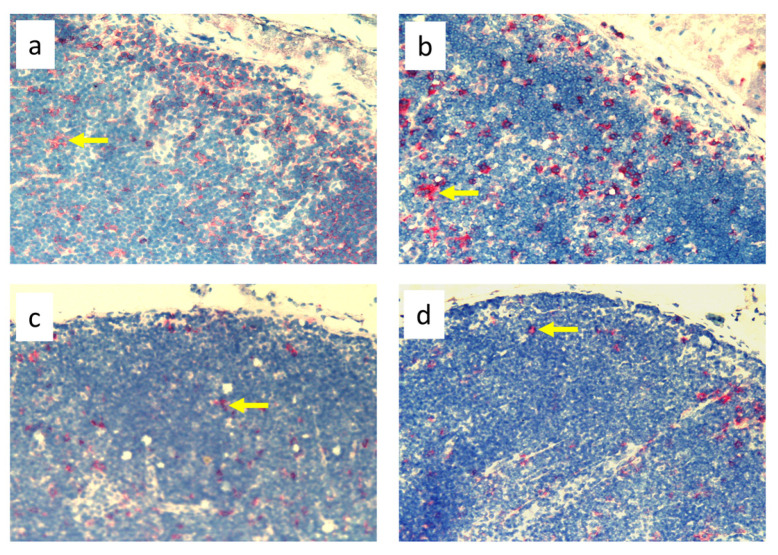
Popliteal lymph nodes stained with antibody against dendritic cells (OX62^+^) on experimental day 9. (**a**). Normal node (7 × 0.9% NaCl). (**b**). 7× infection with *S. epidermidis*. (**c**). Ligation of afferent lymphatics and 7 × 0.9% NaCl. (**d**). Ligation of afferent lymphatics and 7× infection with *S. epidermidis*. Arrows indicate positively stained cells. Magnification, 200×. (**a**,**b**) as in Cąkała-Jakimowicz et al., 2022 [10], with permission.

**Figure 6:**
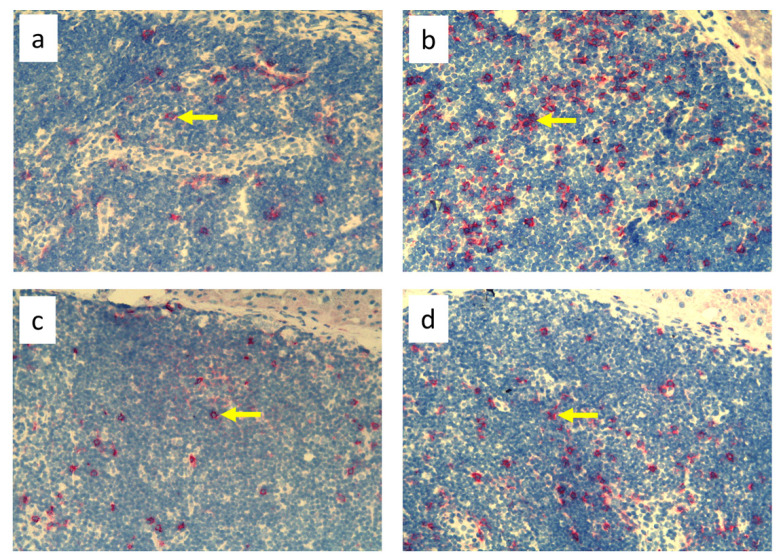
Popliteal lymph nodes stained with antibody against granulocytes (HiS48^+^) on day 9 of the experiment. (**a**). Normal node (7 × 0.9% NaCl). (**b**). 7× infection with *S. epidermidis*. (**c**). Ligation of afferent lymphatics and 7 × 0.9% NaCl. (**d**). Ligation of afferent lymphatics and 7× infection with *S. epidermidis.* Arrows indicate positively stained cells. Magnification, 200×. (**a**,**b**) as in Cąkała-Jakimowicz et al., 2022 [10], with permission.

**Figure 7:**
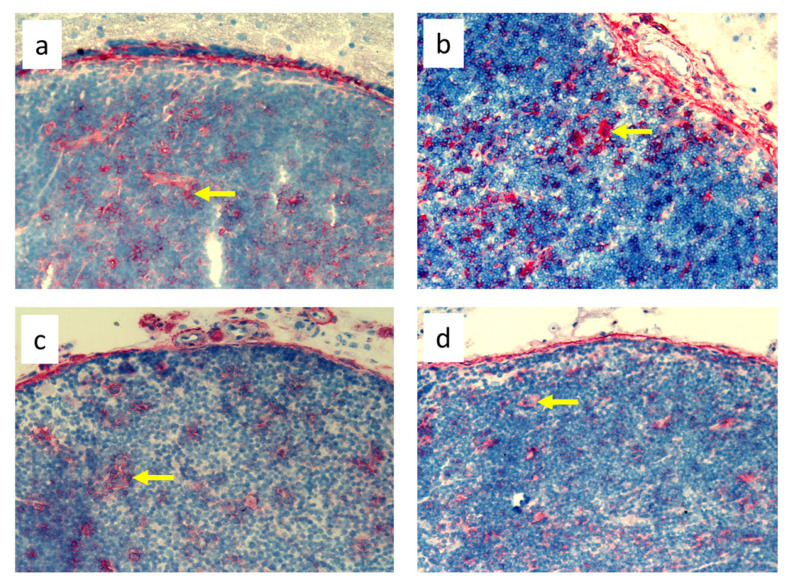
Popliteal lymph nodes stained with antibody against stem cells, thymocytes, and immature B cells (CD90^+^) on day 9 of the experiment. (**a**). Normal node (7 × 0.9% NaCl). (**b**). Seven-times infection with *S. epidermidis*. (**c**). Ligation of afferent lymphatics and 7 × 0.9% NaCl. (**d**). Ligation of afferent lymphatics and 7× infection with *S. epidermidis.* Arrows indicate positively stained cells. Magnification, 200×.

**Figure 8:**
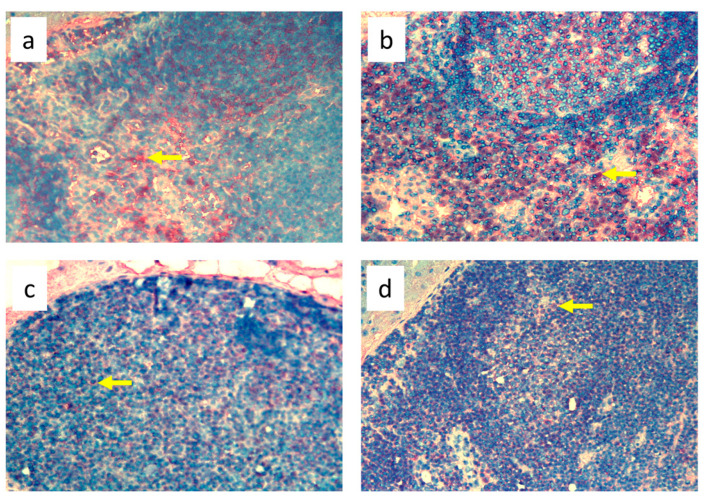
Popliteal lymph nodes stained with antibody against B cells (CD19^+^) on day 9 of the experiment. (**a**). Normal node (7 × 0.9% NaCl). (**b**). 7× infection with *S. epidermidis*. (**c**). Ligation of afferent lymphatics and 7 × 0.9% NaCl. (**d**). Ligation of afferent lymphatics and 7× infection with *S. epidermidis*. Arrows indicate positively stained cells. Magnification, 200×.

Analysis of immunohistochemical images using the ImageJ counting tool showed that, in uninfected animals, lymphedema itself resulted in a reduction in the number of OX62^+^ dendritic cells in the popliteal lymph nodes compared to normal lymph flow. However, in animals infected with *S. epidermidis* and with disturbed lymph flow, we detected a decrease in the density of CD68^+^ macrophages and monocytes, CD19^+^ B lymphocytes, OX62^+^ dendritic cells, HiS48^+^ granulocytes, and CD90^+^ stem cells, thymocytes, and immature B cells compared to animals infected with *S. epidermidis* but with normal lymph flow. We also revealed a decrease in the density of CD4^+^ T helper lymphocytes and monocytes in the nodes of *S. epidermidis*-infected animals with both normal and impaired lymph flow compared to uninfected groups, which coincides with cytometric evaluation. We observed no differences in the densities of the other cell subpopulations examined (Figure 3, Figure 4, Figure 5, Figure 6, Figure 7 and Figure 8, Supplementary Table S2).

### 3.3. Quantitative Changes in Cell Subpopulations in the Skin

**Figure 9:**
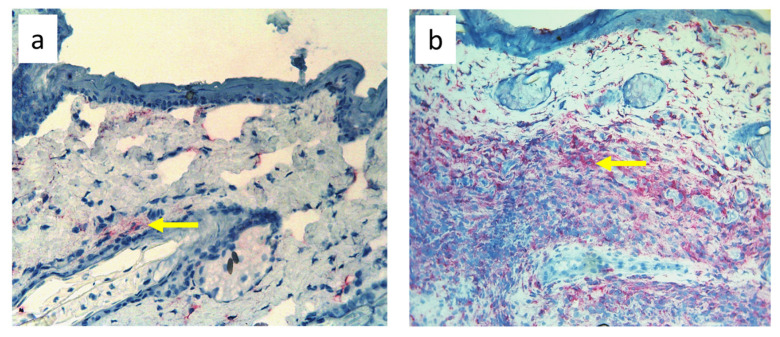
Paw skin stained with antibody against MHC class II antigen-presenting cells (OX6^+^ dendritic cells, macrophages, and lymphocytes). (**a**). Normal lymph flow and 7× infection with *S. epidermidis*. (**b**). Ligation of afferent lymphatics and 7× infection with *S. epidermidis*. Arrows indicate positively stained cells. Magnification, 200×.

**Figure 10:**
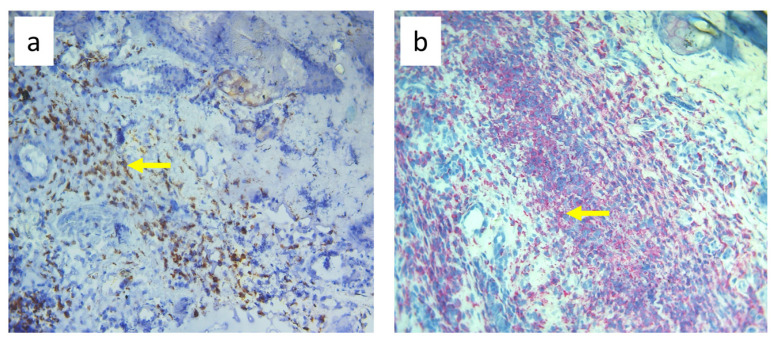
Paw skin stained with antibody against CD68^+^ cells (macrophages and monocytes). (**a**). Normal lymph flow and 7× infection with *S. epidermidis*. (**b**). Ligation of afferent lymphatics and 7× infection with *S. epidermidis.* Arrows indicate positively stained cells. Magnification, 200×.

**Figure 11:**
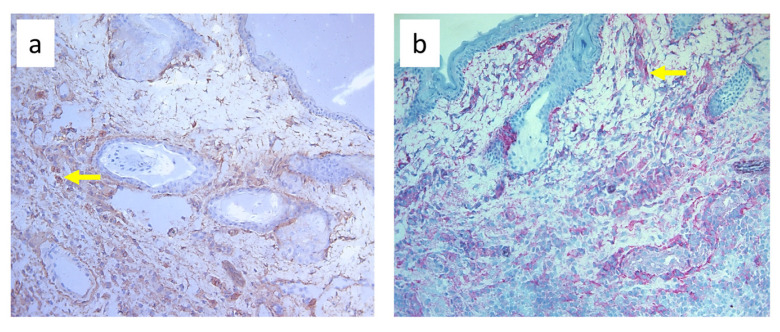
Paw skin stained with antibody against CD90^+^ cells (stem cells, thymocytes, and immature B cells). (**a**). Normal lymph flow and 7× infection with *S. epidermidis*. (**b**). Ligation of afferent lymphatics and 7× infection with *S. epidermidis*. Arrows indicate positively stained cells. Magnification, 200×.

Nine-day edema alone resulting from the impaired lymph flow did not affect immune cell content in the skin. Infiltration of MHC class II antigen-presenting cells (OX6^+^), which include activated dendritic cells, macrophages, and lymphocytes, both directly under the epidermis and in the deeper layers of the dermis, was denser in *S. epidermidis*-infected animals with impaired lymph flow than in those with normal lymph flow (Figure 9, Supplementary Table S3). Moreover, lymph stasis in infected animals resulted in the appearance of denser infiltrates of macrophages and monocytes (CD68^+^) and stem cells, thymocytes, and immature B cells (CD90^+^) (Figure 10 and Figure 11, Supplementary Table S3).

## 4. Discussion

Infection of the skin of a rat limb with *S. epidermidis* combined with impaired lymph flow reflects the significant clinical problem of lymphedema of the limb complicated by chronic infection with saprophytic bacterial flora. *S. epidermidis* was selected for these experiments because we cultured *S. epidermidis* in 10 out of 28 skin biopsies obtained from patients with leg lymphedema. Moreover, we observed numerous infiltrates of CD1a^+^ Langerhans cells and MHC class II antigen-presenting cells (HLA-DR^+^) in these biopsies, which indicates activation of the immune response (unpublished data). In addition, in our previous studies of the impact of infection with selected strains of cutaneous saprophytes on the immune cell response, *S. epidermidis* infection resulted in the highest number of perivascular infiltrates in rat skin [30].

Here, we found that damage to the lymphatic drainage in the limb itself, resulting in 9 days of edema, did not cause changes in the weight of the popliteal lymph nodes or the number of cells in the nodes compared to the control group without edema [10]. However, *S. epidermidis* infection superimposed on the edema significantly increased the mass and cellular content of the nodes. Nonetheless, we did not demonstrate significant differences in the mass and number of cells in the nodes between groups of infected animals with either normal or impaired lymph flow. This observation suggests that the main factor mobilizing the immune system is the infection itself, at least in the first few days of edema.

Cytometric evaluation of cells isolated from popliteal nodes showed that, in comparison to normal lymph flow and lack of saprophyte infection [10], lymphedema itself and *S. epidermidis* infection with or without lymph stasis significantly reduced the percentage of CD4^+^ T helper lymphocytes and monocytes. In addition, in animals with lymphedema and *S. epidermidis* infection, we observed less activated CD4^+^ MHC class II^+^ T helper lymphocytes. In a mouse model of lymphedema, naïve CD4^+^ T lymphocytes are activated in draining lymph nodes after interacting with antigen-presenting dendritic cells and then migrate to the skin, promoting fibrotic and inflammatory processes and inhibiting lymphangiogenesis and all functions of the lymphatic system [31]. The findings of Ly et al. suggest that damage to the lymphatic system results in the expansion of the population of CD4^+^ T lymphocytes in edematous tissues and that even a small number of these cells is sufficient to cause inflammation [20]. Avraham et al. showed that the number of CD4^+^ T lymphocytes infiltrating the tissue is positively correlated with the severity of the edema in humans [32], and studies in mice have shown that neutralization of CD4^+^ T lymphocytes is a way to prevent and treat lymphedema [33,34]. In addition, in bacterial infections in peripheral tissues, many proliferating CD4^+^ T lymphocytes leave the draining lymph nodes and migrate to the infected tissue through the bloodstream [35]. It seems that, in the initial stage of the disease, both the swelling and the constantly penetrating saprophytic bacterial flora may intensify the migratory processes of activated CD4^+^ T helper lymphocytes from the draining lymph nodes to the skin, which may favor inflammation in the edematous area and exacerbate the course of the disease, leading to further consequences, namely, tissue fibrosis and fat deposition.

Despite the lack of differences in cell mass and number in the nodes of *S. epidermidis*-infected animals with or without edema, we showed, using immunohistochemical staining of nodes, that impaired lymphatic transport not only led to excessive accumulation of lymph in the limb but also contributed to a reduction in the transport of specific types of immunologically competent cells from swollen skin to draining lymph nodes. Namely, we showed a fall in the number of OX62^+^ dendritic cells, HiS48^+^ granulocytes, CD68^+^ macrophages and monocytes, CD90^+^ stem cells, thymocytes, immature B cells, and CD19^+^ B cells in the nodes of animals with edema and infected with *S. epidermidis* compared to infected animals with normal lymph flow (Figure 3, Figure 4, Figure 5, Figure 6, Figure 7 and Figure 8, Supplementary Table S2). In contrast, in the skin of animals with edema and infected with *S. epidermidis*, we observed numerous infiltrates, located directly under the epidermis and in the deep layers of dermis, of MHC class II antigen-presenting cells (which include activated macrophages, dendritic cells, and lymphocytes), CD68^+^ macrophages and monocytes, and CD90^+^ stem cells, thymocytes, and immature B cells, and these infiltrates were larger than in infected animals without lymph stasis (Figure 9, Figure 10 and Figure 11, Supplementary Table S3). MHC class II antigens are expressed on the surface of antigen-presenting cells and are presented to antigen-specific CD4^+^ T helper lymphocytes [36]. In an infection, bacterial antigen constantly penetrates, and a defective transport of immune cells involved in the antibacterial response, as well as the accumulation of innate immune cells, such as monocytes, macrophages, dendritic cells, and granulocytes, may disturb the protection processes and enhance inflammation in peripheral tissue.

These observations suggest a complex role for macrophages in the pathophysiology of lymphedema. They affect the deposition of fibro-adipose tissue, lymphangiogenesis, and the pumping work of lymphatic vessels, mainly by secreting various growth factors and cytokines that affect lymphatic endothelial cells [4]. It should be noted, however, that skin macrophages are primarily involved in non-specific immune reactions through their ability to migrate, kill, and phagocytose pathogens and damaged or dead cells [37]. The dense infiltration of CD68^+^ macrophages and monocytes in the commensal-infected skin of animals with edema, observed here, indicates the involvement of these cells in the inflammatory process associated with the elimination of *S. epidermidis* (Figure 10, Supplementary Table S3). Resident dermal macrophages are programmed locally during staphylococcal skin infection, leading to transiently increased resistance to secondary infection [38]. A reduction in the percentage of CD68^+^ macrophages and monocytes in nodes draining the infected skin of animals with impaired lymph flow (Figure 3, Figure 4, Figure 5, Figure 6, Figure 7 and Figure 8, Supplementary Table S2) may delay the elimination of the antigen and also impair communication with other cells participating in the antibacterial defense. Lymph node macrophages effectively capture antigens carried by the lymph, present them to B lymphocytes, dendritic cells, and T lymphocytes, and produce trophic factors and cytokines [39].

Ligation of afferent lymphatics resulted in the inability of cells maturing in the skin upon absorbing the antigen to migrate efficiently to the nodes and perform their role in innate immunity in a timely manner. We found numerous infiltrates of cells expressing MHC class II^+^ cells in swollen, *S. epidermidis*-infected skin, especially close to the epidermis (Figure 9, Supplementary Table S3). This is consistent with the observations of Rutkowski et al., who showed slower migration of Langerhans cells through the edematous dermis. These cells persisted in peripheral tissue, produced inflammatory mediators, and intensified the inflammatory process [40,41,42]. Neutrophils entering the nodes through afferent lymphatics display an activated phenotype similar to antigen-presenting cells [43], suggesting that they play an important role in interacting with lymph node immune cells and modulating adaptive immunity. We assume that their reduced number in the nodes draining the swollen skin of animals infected with *S. epidermidis* may, on the one hand, disturb the mechanisms of antibacterial protection, and, on the other hand, exacerbate the inflammatory reaction through their accumulation in the peripheral tissue.

In our study, we also showed intense infiltration of stem cells, thymocytes, and immature B cells expressing the CD90^+^ antigen in the skin of animals with impaired lymph flow and infected with *S. epidermidis* (Figure 11, Supplementary Table S3), with an accompanying reduced number in the draining nodes (Figure 3, Figure 4, Figure 5, Figure 6, Figure 7 and Figure 8, Supplementary Table S2). CD90 expression is tightly regulated during the development of inflammation and fibrosis. Therefore, dense infiltrates in swollen and infected skin may indicate an ongoing strong inflammatory process.

The limitation of our work is the short duration of the experiment, which allowed us to observe the reaction of immune system cells only in the early stages of disturbances in lymph flow. However, we considered our experiments to be a reasonable reflection of the immunological model of primary skin infection with *S. epidermidis* from our previous work [10] and this allowed us to compare data between normal lymph flow and lymphedema. On the other hand, this experimental model is the strong point of our work: although it is not highly invasive, it allowed the observation of immunological processes in nodes and tissues affected by lymph stasis and infection and may therefore be a good model for studying analogous processes occurring in human tissues.

## 5. Conclusions

In edematous skin that is massively penetrated by saprophytic bacterial flora, blocking the migration by afferent lymphatics of specific types of activated innate immune cells may not only cause an exacerbation of the inflammatory reaction in the peripheral tissue (skin) but also delay and complicate the processes of antibacterial protection, which mainly take place in the lymphatic organs, that is, draining lymph nodes. We suggest that in addition to the standard treatment of lymphedema involving massages, lymphatic drainage, intermittent pneumatic compression, and the use of various types of compression garments [44], systematic cleaning of the affected skin, and, if necessary, anti-inflammatory and antibiotic treatment are necessary to prevent saprophytic flora-induced inflammation.

## Figures and Tables

**Table 1:** Experimental groups.

Experimental Group	Procedure
**Interruption of lymphatics** n = 6 rats (12 lymph nodes, 4 paw skin biopsies)	Ligation of afferent lymphatics Daily subepidermal injection of 0.1 mL of 0.9% NaCl into the paw skin for 7 days
**Interruption of lymphatics +** ***S. epidermidis*** **infection** n = 6 rats (12 lymph nodes, 4 paw skin biopsies)	Ligation of afferent lymphatics Daily subepidermal injection of *S. epidermidis* (6 × 10^7^ cells suspended in 0.1 mL of 0.9% NaCl) into the paw skin for 7 days

**Table 2:** Determination of cell phenotypes using flow cytometry and immunohistochemistry.

Cell Type	Antigen (Rat Clone)
T lymphocytes	CD43^+^ (W3/13^+^)
T helper lymphocytes, monocytes	CD4^+^ (W3/25^+^)
T cytotoxic lymphocytes	CD8^+^ (OX8^+^)
B lymphocytes	CD19^+^ (OX12^+^)
Dendritic cells	OX62^+^
Macrophages, monocytes	CD68^+^ (ED1^+^)
Stem cells, thymocytes, immature B lymphocytes	CD90^+^ (OX7^+^)
Granulocytes	HiS48^+^
Activated antigen-presenting cells	MHC class II^+^ (OX6^+^)
ICAM-1, dendritic cells, endothelial cells	CD54^+^
Activated T lymphocytes	CD43^+^ MHC class II^+^
Activated helper T lymphocytes	CD4^+^ MHC class II^+^
Activated cytotoxic T lymphocytes	CD8^+^ MHC class II^+^

## Data Availability

Data are contained within the article and Supplementary Materials.

## Supplementary Materials

The following supporting information can be downloaded at https://www.mdpi.com/article/10.3390/biomedicines11123234/s1: Supplementary Table S1: Antibodies used for flow cytometry and immunohistochemistry; Supplementary Table S2: The mean number ± standard deviation of positively stained cells from four representative fields of view (magnification 400×) of popliteal lymph nodes section; Supplementary Table S3: The mean number ± standard deviation of positively stained cells from three representative fields of view (magnification 400×) of skin sections; Supplementary Figure S1: Examples of flow cytometry gating strategy (the Cell Quest Pro program, Becton Dickinson) for leukocyte populations isolated from popliteal lymph nodes.
