# Supplementary material for: Interruption of Lymph Flow Worsens the Skin Inflammation Caused by Saprophytic Staphylococcus epidermidis
Source: Biomedicines. 2023 Dec 6;11(12):3234. doi: 10.3390/biomedicines11123234 (PMC10740757; doi:10.3390/biomedicines11123234)
Supplement: Supplementary file 1 [file biomedicines-11-03234-s001.zip › biomedicines-2696912-supplementary.pdf]

Supplementary Table S1. Antibodies used for flow cytometry and immunohistochemistry.

CD (antigen)	Rat clone	Manufacturer	Concentration	Dilution
CD43	W3/13	S	1.0 mg/ml	1:100
CD4	W3/25	S	1.0 mg/ml	1:100
CD8	OX8	S	1.0 mg/ml	1:100
CD19 Ig κ-chain	OX12	S	1.0 mg/ml	1:100
-	OX62	S	0.1 mg/ml	1:30
CD68	ED1	S	0.1 mg/ml	1:70-1:100
-	HiS48	S	-	1:100
CD90	OX7	S	1.0 mg/ml	1:100
MHC CLASS II RT1B	OX6	S	1.0 mg/ml	1:100
MHC CLASS II RT1B	OX6 FITC	S	1.0 mg/ml	1:100
CD54 (ICAM-1)	1A29	S	1.0 mg/ml	1:50
IgG1	-	S	0.1 mg/ml	1:10
IgG2a	-	S	0.1 mg/ml	1:10
IgG1 PE	R3-34	BD	0.1 mg/ml	1:10
IgG PE	Poly24030	BL	0.1 mg/ml	1:10
IgG1 FITC	R3-34	BD	0.1 mg/ml	1:10
IgG FITC	HTK888	BL	0.5 mg/ml	1:10

S: Serotec Ltd, Kidlington, United Kingdom; BD: Becton Dickinson Biosciences Pharmingen, San Jose, CA, USA; BL: Bio Legend, San Diego, CA, USA.

Supplementary Table S2. The mean number ± standard deviation of positively stained cells from four representative fields of view (magnification 400×) of popliteal lymph nodes section.

	T helper lymphocytes, monocytes CD4+	Granulocytes HiS48+	Dendritic cells OX62+	Macrophages, monocytes CD68+	Stem cells, immature B cells CD90+
Control	416 ± 86	35 ± 9	168 ± 68	91 ± 19	122 ± 21
S. epidermidis	192 ± 53	138 ± 30	75 ± 13	159 ± 16	196 ± 12
Interruption of lymphatics	354 ± 40	48 ± 22	42 ± 9	122 ± 19	149 ± 25
Interruption of lymphatics, *S. epidermidis*	186 ± 38	48 ± 9	45 ± 12	107 ± 15	114 ± 7

Supplementary Table S3. The mean number ± standard deviation of positively stained cells from three representative fields of view (magnification 400×) of skin sections.

	MHC class II antigen presenting cells (dendritic cells, macrophages, lymphocytes)	Macrophages, monocytes CD68+	Stem cells, immature B cells CD90+
S. epidermidis	108 ± 40	120 ± 33	124 ± 30
Interruption of lymphatics, *S. epidermidis*	222 ± 16	377 ± 19	280 ± 38

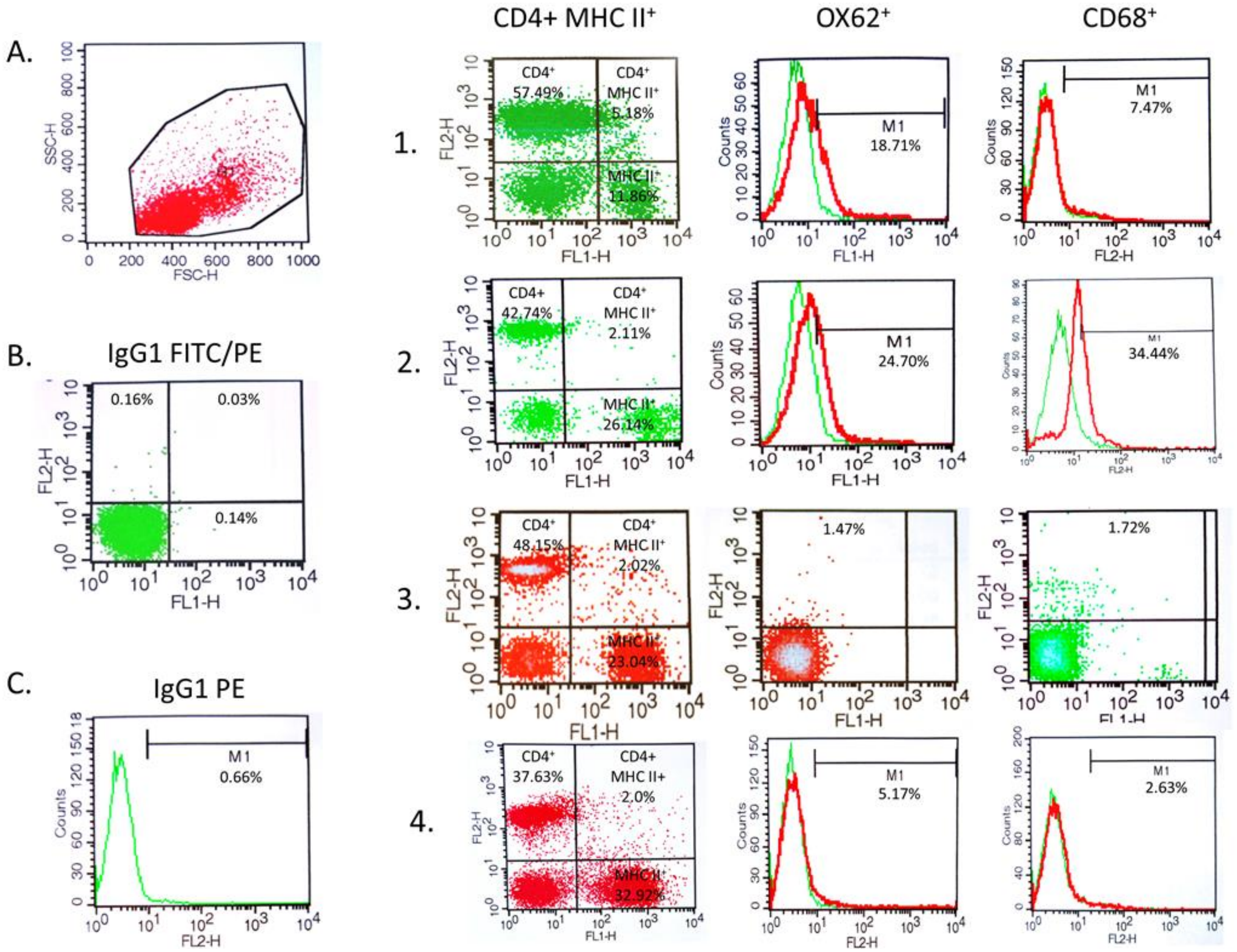

Supplementary Figure S1. Example of flow cytometry gating strategy (the Cell Quest Pro program, Becton Dickinson) for leukocyte populations isolated from popliteal lymph nodes. Dot plots or histograms are presented for the CD4$^+$, MHC II$^+$, CD4$^+$ MHC II$^+$, OX62$^{+,}$ and CD68$^+$ subpopulations. A. Dot plots of physicochemical properties of cells. FSC-H: Forward Scatter Channel high peak. SSC-H: Side Scatter Channel high peak. B. and C. isotypic controls. 1. Control: normal lymph flow and 7 × 0.9% NaCl injection. 2. *S. epidermidis:* normal lymph flow and 7 × infection with *S. epidermidis*. 3. Interruption of lymphatics: lymph stasis and 7 × 0.9% NaCl injection. 4. Interruption of lymphatics and *S. epidermidis*: lymph stasis and 7 × infection with *S. epidermidis.*
